# Lipopolysaccharide induces IFN-γ production in human NK cells

**DOI:** 10.3389/fimmu.2013.00011

**Published:** 2013-01-28

**Authors:** Leonid M. Kanevskiy, William G. Telford, Alexander M. Sapozhnikov, Elena I. Kovalenko

**Affiliations:** ^1^Laboratory of Cell Interactions, Department of Immunology, Shemyakin-Ovchinnikov Institute of Bioorganic Chemistry,Russian Academy of ScienceMoscow, Russia; ^2^Experimental Transplantation and Immunology Branch, National Cancer Institute, National Institutes of HealthBethesda, MD, USA

**Keywords:** NK cells, lipopolysaccharide, cytokine production, cytotoxicity, flow cytometry

## Abstract

Natural killer (NK) cells have been shown to play a regulatory role in sepsis. According to the current view, NK cells become activated via macrophages or dendritic cells primed by lipopolysaccharide (LPS). Recently, TLR4 gene expression was detected in human NK cells suggesting the possibility of a direct action of LPS on NK cells. In this study, effects of LPS on NK cell cytokine production and cytotoxicity were studied using highly purified human NK cells. LPS was shown to induce IFN-γ production in the presence of IL-2 in NK cell populations containing>98% CD56^+^ cells. Surprisingly, in the same experiments LPS decreased NK cell degranulation. No significant expression of markers related to blood dendritic cells, monocytes or T or B lymphocytes in the NK cell preparations was observed; the portions of HLA-DR^-bright^, CD14^+^, CD3^+^, and CD20^+^ cells amounted to less than 0.1% within the cell populations. No more than 0.2% of NK cells were shown to be slightly positive for surface TLR4 in our experimental system, although intracellular staining revealed moderate amounts of TLR4 inside the NK cell population. These cells were negative for surface CD14, the receptor participating in LPS recognition by TLR4. Incubation of NK cells with IL-2 or/and LPS did not lead to an increase in TLR4 surface expression. TLR4^-^CD56^+^ NK cells isolated by cell sorting secreted IFN-γ in response to LPS. Antibody to TLR4 did not block the LPS-induced increase in IFN-γ production. We have also shown that R_e_-form of LPS lacking outer core oligosaccharide and *O*-antigen induces less cytokine production in NK cells than full-length LPS. We speculate that the polysaccharide fragments of LPS molecule may take part in LPS-induced IFN-γ production by NK cells. Collectively our data suggest the existence of a mechanism of LPS direct action on NK cells distinct from established TLR4-mediated signaling.

## INTRODUCTION

Natural killer (NK) cells are the major interferon-gamma (IFN-γ) producers in the early stages of the immune response (Artavanis-Tsakonas and Riley, 2002; Thäle and Kiderlen, 2005). IFN-γ production can be triggered in NK cells as a result of contact and recognition of target cells after activating interactions with other immune cells, particularly with dendritic cells (DC) and macrophages, or under the action of various cytokines, notably IL-12, IL-18, and IL-2, produced at different stages of infection and inflammation (Gerosa et al., 2002; Cooper et al., 2004; Tu et al., 2008).

Natural killer cells possess multiple sensors on their surface for the detection of PAMP (pathogen-associated molecular patterns) and DAMP (damage-associated molecular patterns) caused by infection or cell stress (Chalifour et al., 2004). A number of studies *in vitro* and *in vivo* have shown that NK cells can be activated by lipopolysaccharide (LPS), the component of the outer membrane of Gram-negative bacteria (Goodier and Londei, 2000; Varma et al., 2002). NK cells now seem to be one of the important cell types participating in the septic inflammatory process (reviewed in Chiche et al., 2011; Souza-Fonseca-Guimaraes et al., 2012a). Several studies have demonstrated that LPS can activate NK cells indirectly. LPS primarily activates DC or macrophages through the established LPS receptor TLR4 (Toll-like receptor 4) triggering production of cytokines (IL-12, IL-18) and surface expression of several stimulating ligands in these cells, including B-7 and some NKG2D ligands, leading to NK cell activation (Goodier and Londei, 2000; Gerosa et al., 2002). This model of indirect NK cell activation by LPS is now generally accepted. Alternatively, it has been proposed that LPS directly influences NK cells by engaging TLR4 on the NK cell surface. Several reports suggest that human NK cells express TLRs, particularly, TLR2 and TLR4, at least on the mRNA level (Saikh et al., 2003; Lauzon et al., 2006; Mian et al., 2010; Chiche et al., 2011). Recently intracellular TLR4 expression was shown for NK cells (Souza-Fonseca-Guimaraes et al., 2012b). Direct activating effects of the agonists of TLR2, 3, 7, 8, and 9 on NK cell activity have been demonstrated (Becker et al., 2003; Sivori et al., 2004; Gorski et al., 2006; Lauzon et al., 2006; Sawaki et al., 2007; Toka et al., 2009). Both surface expression (O’Connor et al., 2005) and functional activity (Mian et al., 2010) of TLR4 have also been detected in human NK cells. Collectively, these data favor the hypothesis of both direct and indirect mechanisms for LPS modulation of NK cell activity.

In this study, we investigated the hypothesis of direct action of LPS on NK cells. A stimulating effect of LPS on cytokine-induced IFN-γ production was observed in highly purified fractions of human NK cells isolated by magnetic separation. Increase of IFN-γ production in these experiments corresponded to a decrease in NK cell degranulation in response to K562 target cells. Surprisingly we did not detect any significant surface TLR4 expression in the cells that produced increased amount of IFN-γ. Instead, we demonstrated that these cells were slightly positive for intracellular TLR4. Using flow cytometry multicolor analysis we found only negligible numbers of DC, monocytes, T and B cells within the isolated CD56^+^ cell population. Moreover, NK cells isolated by fluorescence-activated cell sorting (FACS) with intentional exclusion of surface TLR4-positive cells responded well to LPS stimulation. Blocking antibody to TLR4 did not inhibit the LPS-induced increase of IFN-γ production suggesting the existence of a mechanism of LPS activation distinct from established TLR4-mediated signaling.

## MATERIALS AND METHODS

### ISOLATION OF HUMAN NK CELLS AND CULTURE CONDITIONS

Adult volunteers gave informed consent for their blood to be used in this study, which was approved by ethics committees of The Russian State Medical University. Peripheral blood mononuclear cells (PBMC) were isolated from heparinized whole blood by centrifugation on Ficoll gradients with a density of 1.077 g/ml (ICN). Two strategies were used to purify NK cells. First, magnetic separation of NK cells was performed using a NK cell negative isolation kit (Miltenyi Biotec, Bergisch Gladbach, Germany) according to the manufacturer’s protocol using LD columns. The percentage of CD56^+^ cells in the preparations after separation was routinely 95–99% as verified by flow cytometry. Second, CD3^-^CD14^-^CD56^+^TLR4^-^ cells were separated from PBMC fraction by fluorescent-activated cell sorting. The post-sort population purity was always greater than 95%. Isolated NK cells were cultivated in 96 U-well plates (Costar) in an incubator set at 5% CO_2_ and 37°C at cell concentrations of 1.5 × 10^6^ cells/ml in culture medium consisted of RPMI-1640 supplemented with 10% heat-inactivated FCS (HyClone), 2 mM L-glutamine and antibiotic-antimycotic solution (1:100; Sigma-Aldrich, St. Louis, MO, USA) for 18 h. Recombinant human IL-2 purchased from Hoffmann-La-Roche (500 U/ml), recombinant human IL-12 from Sigma-Aldrich (10 ng/ml), LPS from *E. coli* strain 026:B6 (Sigma-Aldrich) or Kdo_2_-lipid A from *E. coli* K15 heptose-deficient strain WBB06 (ENZO Life Sciences) were added to the cell culture. Prior to use the vials containing LPS and lipid A preparations were shaken by vortex mixer for 30 s and sonicated.

### SURFACE FLUORESCENT IMMUNOSTAINING, FLOW CYTOMETRY, AND CELL SORTING

Cell labeling with fluorochrome-conjugated antibodies and subsequent flow cytometry analysis was used to determine the presence and percentage of leukocyte subsets and to characterize antigen surface expression in isolated NK cells. The following conjugated anti-human antibodies were: CD3-FITC (BioLegend, San Diego, CA, USA), CD3-PE-Cy7 (Beckman Coulter, Brea, CA, USA), CD3-PE (Invitrogen Life Technologies, Carlsbad, CA, USA), CD56-APC (Beckman Coulter), CD56-PE (Dako, Glostrup, Denmark), CD14-PE (BD Pharmingen, San Diego, CA, USA), CD20-PerCP (BD Pharmingen), anti-TLR4 and anti-TLR4-FITC (clone HTA125, HyCult Biotech), anti-TLR4 (clone HT52, eBioscience), CD11c-PE-Cy5 (BioLegend), CD141-PE (BioLegend), CD303-APC (Miltenyi Biotec), HLA-DR-FITC (BD Pharmingen), HLA-DR-PE (eBioscience), isotype control IgG2a (eBioscience). Cells were incubated with a premixed antibody cocktail in PBS containing 0.5% BSA and 0.1% sodium azide for 30 min on ice, washed twice and subsequently analyzed by flow cytometry. Samples were analyzed on a FACSCalibur flow cytometer (BD Biosciences, San Jose, CA, USA), equipped with 488 and 640 nm lasers. Multicolor sample analysis and cell sorting were performed on a FACSVantage DiVa cell sorter (BD Biosciences) equipped with 405, 488, and 643 nm lasers and an appropriate set of detectors and filters. Compensation was calculated using the automated compensation algorithm in the DiVa acquisition software. For accurate analysis of rare cell populations no less than 400000 lymphocyte gated events per sample were acquired and analyzed. Cell sorting was carried out at a sheath pressure of 27 psi using a 70-μm nozzle. Cell purities following cell sorting always exceeded 95%.

### TLR4 INTRACELLULAR STAINING

Intracellular staining was performed using a CytoFix-CytoPerm Kit (BD Biosciences) according to manufacturer’s instruction. NK cells were fixed and permeabilized, then labeled with anti-TLR4-FITC (clone HTA125) or isotype control antibody.

### IFN-γ PRODUCTION MEASUREMENT

Cell-free supernatants were collected from unstimulated and stimulated (IL-2, IL-12, or LPS) NK cells after 18 h incubation. Samples were analyzed using an IFN-γ ELISA kit (Vector-Best, Russia) according to the manufacturer’s instruction. Briefly, supernatants were transferred into plate wells coated with immobilized antibody to IFN-γ and incubated for 2 h. After washing (five times) biotinylated antibody to IFN-γ was added for 1 h. After an additional wash streptavidin conjugated with horseradish peroxidase (HRP) was added for 30 min. The chromogenic HRP substrate TMB was added and after 25 min the reaction was terminated with 1 M sulfuric acid. Plates were read using a Multiscan FC plate reader (Thermo Fisher Scientific) set to 450 nm absorption wavelength with reference wavelength of 620 nm.

### ANTIBODY BLOCKING ASSAY

Antibodies to human TLR4 (clone HTA125, BioLegend, and clone HT52, eBioscience) with established blocking activity were used for TLR4 neutralization. NK cells isolated by magnetic separation were preincubated with anti-TLR4 antibody (5 μg/ml) for 40 min at 37°C prior to LPS stimulation. IFN-γ concentrations in supernatants were analyzed by ELISA.

### DEGRANULATION ASSAY

Natural killer cell cytolytic potential was assessed with a degranulation assay based on LAMP-1 (CD107a) cell surface mobilization following NK cell incubation with MHC class I-negative K562 target cells (Alter et al., 2004). In this test, NK cells stimulated for 18 h with IL-2 alone or with IL-2 and LPS (5 μg/ml) were mixed with K562 cells in ratio 1.5:1, centrifuged shortly to start cell-to-cell contact and incubated for 3 h in the presence of PE-Cy5-conjugated anti-human CD107a antibody (eBioscience) and monensin (5 μg/ml, Sigma-Aldrich). Samples without target cells were included to control for spontaneous degranulation. Cells were then washed with PBS containing 0.5% BSA and 0.1% sodium azide and stained with CD3-FITC and CD56-PE antibodies as described above. CD3^-^CD56^+^ cells were then analyzed by flow cytometry, and percentages of CD107a-positive cells were calculated.

### DATA ANALYSIS AND STATISTICS

Experimental data statistical analysis of results was performed using the Student’s unpaired *t*-test. Values of *P* < 0.05 were considered statistically significant. All of the error bars in the graphs represent standard deviations. Flow cytometry data were analyzed using WinMDI version 2.8 (Joe Trotter, Scripps Institute) and FlowJo ver. 7.6 (TreeStar, Ashland, OR, USA) software.

## RESULTS

### LPS INDUCES IFN-γ PRODUCTION IN NK CELLS STIMULATED WITH IL-2

**FIGURE 1:**
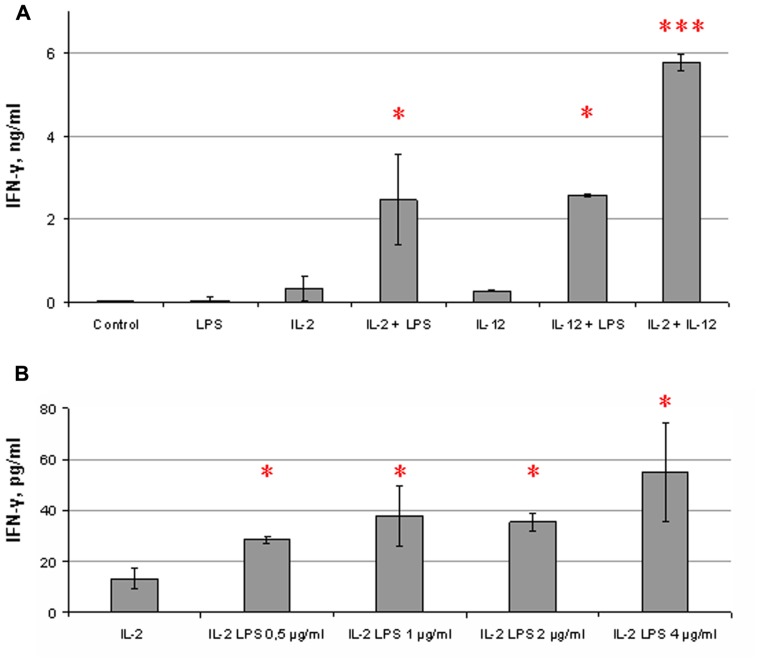
**Co-stimulating influence of LPS on NK cell IFN-γ secretion**. Human NK cells were stimulated for 18 h with cytokines and LPS. Supernatants were harvested and analyzed for IFN-γ by ELISA. Data are mean ± SD of triplicate wells in one representative individual experiment. *P < 0.05, ***P < 0.001, comparisons were shown between cells, stimulated by a cytokine with LPS, and cells, stimulated by the cytokine alone. **(A)** NK cells were incubated in the presence or absence of IL-2 (500 U/ml) or IL-12 (10 ng/ml) and LPS (5 μg/ml). Combination of IL-2 and IL-12 was used as a positive control. **(B)** NK cells were stimulated with IL-2 (500 U/ml) and different doses of LPS. Results of one experiment are shown from the group of experiments demonstrating an increase of IFN-γ secretion under LPS action (12 different donors). Other experiments showed similar effects but differed in IFN-γ level.

We evaluated cytokine production in response to LPS in NK cell populations purified by magnetic separation based on the strategy of lineage-negative NK cell selection. The amount of residual non-NK cells in the population was then measured. Only cell preparations with a high content of CD56^+^ cells (>96%) were used in these experiments. Isolated NK cells were stimulated for 18 h with cytokines (IL-2 or IL-12) and with LPS. IFN-γ contents in culture supernatants were then analyzed by ELISA. It was found that LPS, added to NK cells alone, did not significantly affect IFN-γ production (**Figure 1A**). Addition of LPS simultaneously with IL-2 led to an increase in IFN-γ level. Co-stimulating effects of LPS on IFN-γ production were not restricted to IL-2-induced activation of NK cells. A LPS-mediated increase of IFN-γ production was also found in cultures of NK cells treated with IL-12 indicating that both IL-2 and IL-12 may play accessory role for the LPS action manifestation (**Figure 1A**). Combinations of IL-2 and IL-12 were used as a positive control; IFN-γ level in these samples always exceeded IFN-γ levels in cultures of NK cells stimulated with IL-2 or IL-12 alone. The minimal dose of LPS inducing the IFN-γ elevation in NK cells was 0.5 μg/ml; doses of LPS from 0.5 to 4 μg/ml were found to have perceptible effects on IFN-γ production (**Figure 1B**). Lower doses did not significantly influence the NK cell function. The highest purity of CD56^+^ cells registered in cell preparations responding to LPS by the elevation of IFN-γ production was 99%. It is important to note that the stimulating effect of LPS was not observed in some NK cell preparations. Out of 18 blood samples from healthy donors analyzed, 3 of them did not respond to LPS, 3 samples showed considerable inhibitory effects, and 12 samples demonstrated significant increase of IFN-γ production. There were great inter-individual differences in IFN-γ production; but no clear relationship was observed between initial IFN-γ levels and responses to LPS.

### CHARACTERIZATION OF ISOLATED NK CELL PREPARATIONS

**FIGURE 2:**
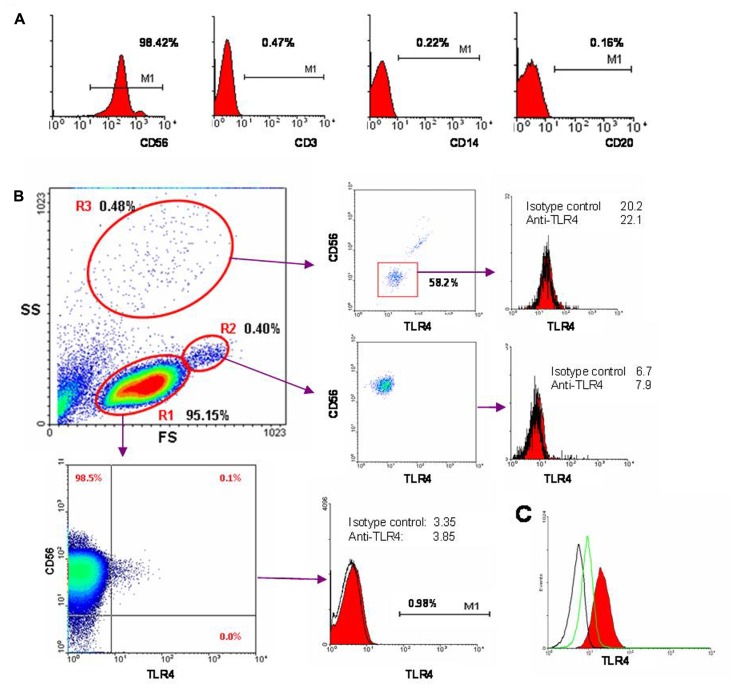
**Cytometric analysis of NK cell population purity and expression of TLR4**. **(A)** Freshly isolated NK cells were stained with fluorochrome-labeled antibodies CD3-FITC, CD14-PE, CD20-PerCP, and CD56-APC and analyzed by flow cytometry. **(B)** Here is shown one of typical results from 18 blood samples used in these experiments. Expression of TLR4 was measured using anti-TLR4-FITC antibody (clone HTA125). Forward scatter (FS) versus side scatter (SS) is presented. Three regions of live cells can be distinguished based on morphology. R1 region (95.15% of all events) consists of CD3^-^CD56^+^ NK cells (98.6%). CD56^-^ cells do not express TLR4. A small population of CD56^+^TLR4^dim^ cells (±0.1%) is detected. On the histogram TLR4 level and isotypic control are shown. R2 region (0.4%) consists of NK cells doublets. R3 region (0.48%) corresponds to granulocytes. **(C)** Intracellular TLR4 expression in NK cells. Black line – autofluorescence control, green – isotype control, red – fixed cells, labeled with anti-TLR4-FITC.

A critical factor of our experiments was the purity of isolated NK cell populations with minimal contamination by other cells types. This was particularly important to exclude cells expressing TLR4, a potential receptor for LPS. Detailed flow cytometric analysis of cell fractions was routinely performed after each NK cell magnetic separation (**Figure 2**). As a rule these cell preparations contained minimal amounts of CD3^+^, CD14^+^, and CD20^+^ cells, with the percentages for these subsets not exceeding 0.1% (**Figure 2A**). Analysis of TLR4 expression was performed to identify in the NK cell preparations the cell types responding to LPS stimulation. An example of a typical cell preparation analysis is presented in **Figure 2B**. In the data forward and side scatter plot three regions can be distinguished: R1 – the usual lymphocyte region; R2 – the usual monocyte region; and R3 – the usual granulocyte region. R1 region consisted almost entirely of CD56^+^ NK cells (97–99%). Virtually no cells in this region expressed detectable TLR4. CD56^-^ cells in this region were all TLR4-negative. In some cases we detected a very small population (not more than 0.2%) of CD56^+^TLR4^+^ cells in this region. CD56^bright^ cells were usually TLR4-negative. The majority of cells in R2 region was CD56^+^ and represented doublets. Granulocytes (R3 region) composed 0.05–2% of all registered events in different samples; they usually did not exceed 0.5%. Most of the cells in R3 region were CD56-negative and were also negative for TLR4. Importantly, the stimulating effects of LPS did not depend on the amount of cells in the sample detected in the granulocyte region. Together, these data showed that there were no cell populations with high expression of TLR4 receptor in the isolated NK cell preparations. It should be noted that after incubation of NK cells with IL-2 and/or LPS the TLR4 surface expression level did not substantially change (data not shown).

**FIGURE 3:**
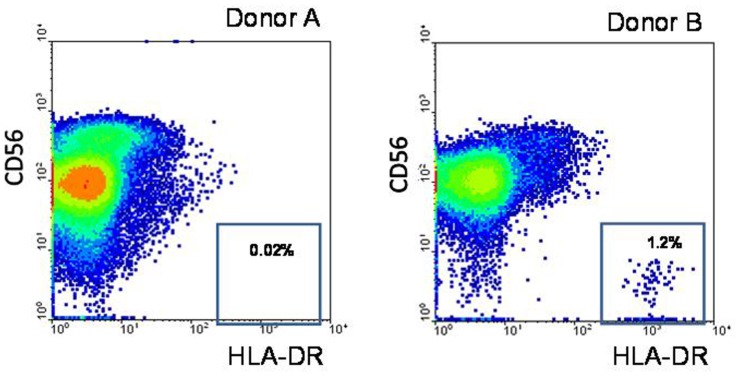
** Expression of HLA-DR was analyzed in different cell samples**. Freshly isolated NK cells were stained with fluorochrome-labeled antibodies CD56-APC and HLA-DR-FITC and analyzed by flow cytometry. *Donor A*: one of typical NK cell preparations with very small amount of HLA-DR^bright^ blood DC cells. *Donor B*: NK cell preparation containing a fraction of HLA-DR^bright^ blood DC cells.

Recently, CD56 weakly-positive populations have been identified among myeloid DCs (Milush et al., 2009). We discriminated DCs in the isolated NK cell preparations using HLA-DR expression. In a few preparations we had detected HLA-DR^bright^ cell population indicating contamination of the samples by DCs (**Figure 3**). Expression of the plasmacytoid and myeloid blood DC subpopulation markers CD303, CD141, CD1c was also analyzed by flow cytometry. As a rule these cells were not detected in the NK cell preparations (data not shown). NK cells stimulated by IL-2 were stained intracellularly with anti-TLR4 antibody. It was shown that although NK cells were not positive for surface TLR4, they expressed intracellular TLR4 (**Figure 2C**).

### EFFECTS OF LPS ON IFN-γ PRODUCTION OF NK CELLS ISOLATED BY CELL SORTING

**FIGURE 4:**
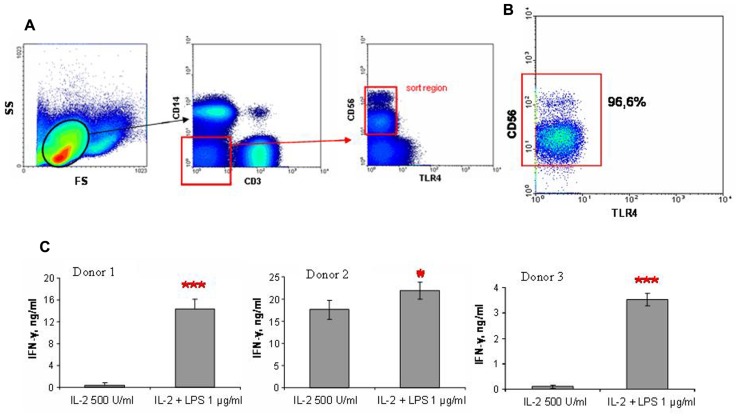
**NK cells were stained with fluorochrome labeled antibodies and sorted using FACSVantage machine**. **(A)** Sort scheme is presented. Gate for lymphocytes was used, then cells were negatively gated for CD3 and CD14, than TLR4-negative CD56^+^ cells were chosen for sorting. **(B)** Post-sort purity of CD56^+^ cells was no less than 95%, no TLR4-positive cells were detected. **(C)** Histograms represent three independent experiments with NK cells from three donors. NK were isolated by cell sorting, than stimulated with IL-2 (500 U/ml) and LPS (1 μg/ml) in duplicate wells. Supernatants were harvested and analyzed for IFN-γ by ELISA. Data are mean ± SD, *P < 0.05, ***P < 0.001.

To determine the role of TLR4 in LPS-induced IFN-γ secretion by NK cells, we performed FACS of CD56^+^ NK cells not expressing TLR4. Blood samples for this series of experiments were collected from donors whose NK cells responded to LPS by increase of IFN-γ production. PBMC isolated by centrifugation using Ficoll gradients were stained with antibodies to CD3, CD14, CD56, and TLR4. CD3^-^CD14^-^CD56^+^TLR4^-^ cells were then separated by FACS (**Figure 4**). Subsequent flow cytometric analysis of sorted cell fractions showed that sorted cells were all TLR4-negative. It is important to note that these cell populations were negative for CD14, a known co-receptor for TLR4. Cells collected after sorting were resuspended in culture medium and stimulated with 500 U/ml IL-2 and 1 μg/ml LPS as described above. The magnitude of the LPS effects differed again between donors. At the same time the samples of sorted NK cells demonstrated higher level of IFN-γ production in response to LPS stimulation in comparison with magnetically separated NK cells. This difference may be connected with additional activation of antibody-labeled NK cells during the sorting procedure. These results demonstrated that LPS caused enhanced IFN-γ production by NK cells independently of surface TLR4 and CD14 expression.

### EFFECTS OF LPS ON NK CELL CYTOTOXICITY

Cytotoxic activity of NK cells stimulated by LPS and IL-2 was estimated in a number of NK cell preparations using a LAMP-1 release assay. Surprisingly, in cell samples where LPS caused an increase of IFN-γ production, LPS treatment resulted in a decrease of K562-dependent NK cell degranulation (**Table 1**).

**Table 1:** Divergent effects of LPS on NK cell cytokine production and cytotoxicity.

	IFN-γ production (pg/ml)			Degranulation (% CD107a^+^ cells)		
	IL-2	IL-2 + LPS	*P*-value	IL-2	IL-2 + LPS	*P*-value
Exp. 1	59.0 ± 5.7	130 ± 24	0.027	20.2 ± 0.4	14.2 ± 1.6	0.008
Exp. 2	2.0 ± 3.5	8.8 ± 0.9	0.040	30.9 ± 1.7	21.1 ± 0.5	0.016
Exp. 3	0	398 ± 343	0.028	45.3 ± 0.9	27.6 ± 3.5	<0.001

*Natural killer cells were stimulated by IL-2 (500 U) alone or by combined treatment with IL-2 and LPS (5 μg/ml) for 18 h. Cytolytic activity of NK cells was estimated by LAMP-1 degranulation assay. IFN-γ levels in supernatants were measured by ELISA. Data are mean ± SD of triplicate wells in individual experiments.*

### BLOCKING ANTIBODIES TO TLR4 DO NOT INHIBIT LPS-INDUCED IFN-γ PRODUCTION IN NK CELLS

**FIGURE 5:**
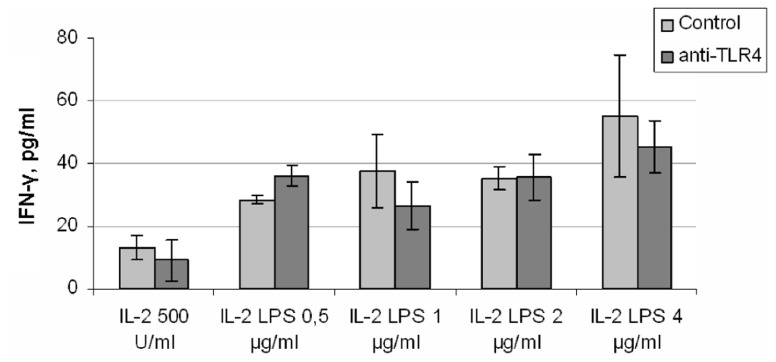
**NK cells isolated by magnetic separation were preincubated with anti-TLR4 antibody (clone HTA125; 5 μg/ml) for 40 min 37°C, than stimulated by IL-2 (500 U/ml) and different doses of LPS**. Supernatants were harvested and analyzed for IFN-γ by ELISA. Data are mean ± SD from one of three experiments with similar results.

Another approach to investigation of the role of TLR4 in LPS-induced IFN-γ production by natural killer cells is to block the TLR4 receptor with a specific blocking antibody. In these experiments we used an anti-human TLR4 antibody with established blocking activity (clone HTA125; Wang et al., 2001, 2003). Anti-TLR4 antibody was added to freshly isolated (by magnetic separation) NK cells in culture medium. Cells were incubated for 40 min with IL-2 (500 U/ml) and LPS in different doses (from 0.5 to 4.0 μg/ml) without washing of NK cells from antibody. Incubation conditions were the same as described above. According to the literature (Wang et al., 2001, 2003) an antibody concentration of 5 μg/ml should lead to at least half-inhibition of LPS-induced cytokine production. However, addition of the antibody did not change IFN-γ level at any LPS dose (**Figure 5**). Higher concentrations of antibody (20 and 50 μg/ml) were checked in similar experiment conditions. This treatment led to a non-specific decrease in IFN-γ production in all samples (data not shown). These experiments were repeated using another inhibitory anti-TLR4 antibody (clone HT52; Tsukamoto et al., 2012), again with no inhibition of the LPS effect (data not shown). Thus, blocking antibodies to TLR4 did not inhibit LPS-induced IFN-γ production in NK cell preparations.

### COMPARISON OF THE EFFECTS OF LPS AND LIPID A ON IFN-γ PRODUCTION BY NK CELLS

**FIGURE 6:**
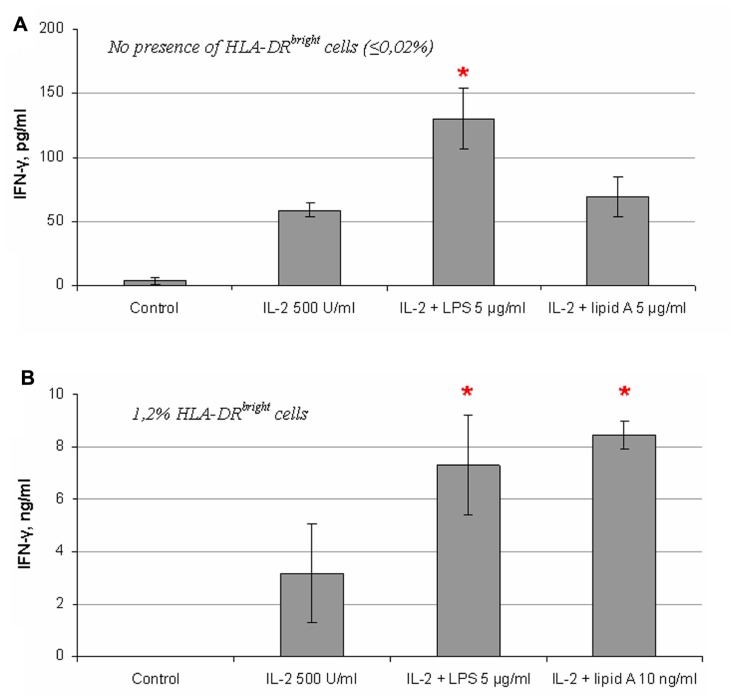
**Comparison of effects of S- and R_**e**_-forms of LPS on IFN-γ production by NK cells**. **(A)** R_e_-form of LPS (lipid A) does not alter significantly IFN-γ production in NK cells at the same concentration as the S-form (full-length LPS; 5 μg/ml). Data from one of four experiments with similar results are presented. **(B)** Significant increase of IFN-γ secretion is induced by low dose of lipid A (10 ng/ml) in NK cell preparation containing a fraction of HLA-DR^bright^ blood DCs (donor B, **Figure 3**).

It is known that LPS interacts with TLR4 via the lipid A part of the LPS molecule. Mutant forms of LPS lacking *O*-antigen or both *O*-antigen and the core oligosaccharide residues but with an intact lipid A region have been shown to intensively induce cytokine production in immune cells through the same signaling pathway as the full-length LPS molecule (Ha et al., 1985; Tamai et al., 2003). To analyze the role of the lipid A fragment in the LPS effects on NK cell activity, we compared the S-form of LPS (whole LPS molecule) and the R_e_-form (lipid A) on NK cell IFN-γ production. The molecular mass of the R_e_-form of LPS is approximately four times less than the whole LPS molecule and therefore the same concentration of R_e_-LPS contains four times more of endotoxin molecules. It is therefore possible that the relative magnitude of the response of NK cells to R_e_-form would be higher than full length LPS molecule based on concentration alone. But in most cases the effect of lipid A was undetectable or insignificant (**Figure 6A**). However in a few experiments we found stimulating effects of R_e_-form at the same dose as S-form (5 μg/ml) (data not shown). In contrast, when we analyzed the effect of these LPS forms on IFN-γ production in NK cell preparation containing blood DC cells highly positive for HLA-DR (**Figure 3B**), the R_e_-form had a stimulating effect at doses as small as 10 ng/ml (**Figure 6B**) in accordance with published data (Gerosa et al., 2002). These results suggest that the lipid A fragment is less important for direct action of LPS on NK cells, and that the polysaccharide portion of the LPS molecule takes part in NK cell activation.

We can therefore summarize, that: (1) NK cells expressed very low levels of TLR4 (or did not express it at all); (2) purified NK cells, with minimal non-NK cells presence, responded to LPS by increasing IFN-γ production; (3) sorted CD14^-^TLR4^-^ NK cells responded well to LPS stimulation; (4) blocking antibody to TLR4 did not inhibit LPS-induced IFN-γ production; and (5) there were differences between effects of S-form and R_e_-form of LPS on NK cell activity.

## DISCUSSION

Previous studies have shown that NK cells take part in the systemic inflammatory process during of sepsis (Varma et al., 2002; Chiche et al., 2011; Souza-Fonseca-Guimaraes et al., 2012a). LPS-stimulated DCs and macrophages activate NK cells using cytokines and contact interactions. On the other hand, IFN-γ produced by NK cells is a potent activator of DCs and macrophages. Thus, NK cells are among the major multipliers of systemic inflammatory response in sepsis. However, additional investigations of LPS influence on NK cell activity are necessary for better understanding of the role of these cells in sepsis pathogenesis. Here we demonstrate that LPS can directly stimulate IFN-γ production in human NK cells. This effect appears to be independent of TLR4, a known receptor for LPS.

First of all, we have found that highly purified NK cells isolated by magnetic separation from human peripheral blood of healthy volunteers respond to LPS in the presence of activating cytokines IL-2 and IL-12 (**Figure 1**). Significant co-stimulating effects of LPS on IFN-γ production were detected at doses of 0.5–5 μg/ml. However, one-third of samples did not respond to LPS by elevation of IFN-γ production. LPS effects on NK cells have been recently reported in a few publications. LPS was shown to have a weak stimulating effect on IFN-γ production in purified human NK cells (Lauzon et al., 2006; Mian et al., 2010). It should be noted that there are significant difference in experimental conditions between the previous works and the present study. The previous studies used lower concentration of IL-2 (50 U/ml), longer time of incubation (48 h), lower doses of LPS (100 ng/ml) and different cell concentrations (2 × 10^6^/ml). We did not detect any effect of LPS on NK cells without or with low doses of IL-2 (100 U/ml). The present study nevertheless demonstrated that the optimal conditions for LPS-induced IFN-γ production by NK cells can be found. The co-stimulating effect of LPS on cytokine-activated NK cells was in fact reported in another recent work (Souza-Fonseca-Guimaraes et al., 2012b). Authors compared the influence of LPS on NK cells in healthy donors and patients with sepsis and systemic inflammatory response syndrome. In contrast to the healthy donors, NK cells of the patients did not display an increase in IFN-γ production in response to LPS. We speculate that LPS unresponsiveness of some donors observed in our study cohort may be explained by changes in the functional status of NK cells due to some unknown inflammation-related conditions in the donor medical history.

The purity of NK cells used in these previous studies may not be sufficient to clearly demonstrate that LPS acts directly on NK cells. One group of authors reported 3–5% of CD56^-^ cells in their NK cell preparations (Lauzon et al., 2006), while whole blood samples were used in another study (Souza-Fonseca-Guimaraes et al., 2012b). In our work we have performed careful cytometric analysis of isolated NK cell populations, demonstrating the exclusion of contaminating cell types (**Figure 2**). The elevation of IFN-γ production in response to LPS has been demonstrated in the sample containing 99% of CD56^+^ cells.

A critical factor in the present investigation was the number of TLR4-positive cells within the isolated cell preparations. We found virtually no TLR4 expression in non-NK cells. This strongly suggests that LPS can act only through NK cells in our studies. However, analysis of TLR4 expression on NK cells has shown that these cells are also mostly negative for TLR4 as well. Only very small populations of TLR4^dim^ NK cells were detected in a few experiments. This is in contrast to a previous study, which reported that CD56^dim^ NK cells were all TLR4-positive (O’Connor et al., 2005). Nevertheless, in another study (Tadema et al., 2011), only about 0.3–0.5% human NK cells express TLR4, in good agreement with our data. Importantly, stimulation of NK cells with IL-2 and/or LPS did not lead to significant increase of TLR4 expression. In the present study, LPS therefore appears to act on cells displaying no surface TLR4 or expressing considerably lower levels of the receptor comparing with monocytes or neutrophils. Further evidence for the independence of LPS action on surface TLR4 was our demonstration that sorted CD3^-^CD14^-^CD56^+^TLR4^-^ cells respond well to LPS (**Figure 4**). Finally, experiments with blocking antibody to TLR4 also showed that TLR4 did not seem to mediate the LPS induced IFN-γ production by NK cells. However, intracellular staining with anti-TLR4 antibody revealed considerable amounts of TLR4 inside NK cells (**Figure 2C**). Intracellular expression of TLR4 in NK cells has been recently reported (Souza-Fonseca-Guimaraes et al., 2012b). However, increased TLR4 expression in NK cells in patients with sepsis was accompanied by a reduced IFN-γ response to LPS. The phenomenon of intracellular TLR4 expression in NK cells does not fully exclude the possibility of TLR4 participation in NK cell response to LPS. Previous studies have proposed a role for TLR4 traffic between the Golgi apparatus and the plasma membrane (Latz et al., 2002; Husebye et al., 2006). The authors reported a pool of TLR4 molecules in the Golgi and suggested the participation of the intracellular TLR4 in LPS signaling. Additionally, intracellular interaction of LPS with TLR4 in the Golgi was detected in intestinal epithelial cells (Hornef et al., 2003). A recent study also demonstrated that LPS can be recognized by intracellular TLR4 in macrophages (Shibata et al., 2011). These findings suggest that LPS can use an alternative, intracellular pathway. However, experimental evidence for this hypothesis remains limited. According to our data, the R_e_-form of LPS acts on NK cells by a different mechanism than the full-length LPS molecule. This may indicate that the LPS effects are less dependent on lipid A fragment and may be mediated at least in part by polysaccharide fragment of LPS.

In our study, LPS treatment resulted in a remarkable decrease of NK cell degranulation in response to K562 target cells, inversely proportional to IFN-γ production. The divergence of NK cell functional activities distinguishes the mechanism of direct LPS action on NK cells from the well-known mechanism of indirect effects of LPS via secretion of multiple cytokines in antigen-presenting cell populations of the innate immune system (Gerosa et al., 2002; Cooper et al., 2004; Tu et al., 2008). Previously the differences in pathways leading to cytokine production and cytotoxicity have been described for several signaling molecules (Hesslein et al., 2006; Rajagopalan et al., 2006). It is also possible that under different conditions the same molecules can perform different functions. LPS may provide differential regulation of NK cell activity depending on the microenvironment.

It is also possible that LPS interacts with receptors on the NK cell surface other than TLR4. Recently, CD6, the scavenger receptor of the cysteine-rich superfamily, has been shown to bind LPS resulting in activation of the MAPK signaling cascade (Sarrias et al., 2007). CD6 surface expression has also been detected on of CD56^dim^ NK cell subpopulation (Braun et al., 2011). Stimulation of the CD6 receptor by specific antibody cross-linking leads to cytokine secretion but not to cell degranulation. Interestingly, IL-2 was shown to play a co-stimulatory role in the CD6-mediated signaling.

In summary, we have found that LPS can stimulate IFN-γ production in NK cells, but the mechanism of its action remains unclear. Surface TLR4, a known LPS receptor, seems to play no role in this process.

## Conflict of Interest Statement

The authors declare that the research was conducted in the absence of any commercial or financial relationships that could be construed as a potential conflict of interest.

## Acknowledgments

This work was supported by Ministry of Education and Science of the Russian Federation (grant #16.740.11.0200), Programs of Russian Academy of Sciences “Molecular and Cellular Biology” and “Fundamental Investigations of Nanotechnologies and Nanomaterials.”
